# Supplementary material for: Multifunctional cationic nanosystems for nucleic acid therapy of thoracic aortic dissection
Source: Nat Commun. 2019 Jul 18;10:3184. doi: 10.1038/s41467-019-11068-1 (PMC6639375; doi:10.1038/s41467-019-11068-1)
Supplement: Supplementary file 1 — Supplementary Information [file 41467_2019_11068_MOESM1_ESM.pdf]

Supplementary Information for

Multifunctional cationic nanosystems for nucleic acid therapy of thoracic aortic dissection

Xu et al.

Supplementary Table 1 | Characterization of polymers and complexes

Samples	Volume of GMA (mL)	M_n (g mol^{-1})[a]	PDI[a]	Mass ratio of Gd^{3+} (%)[b]	Average PDI[c] (at different N/P ratios)			
					5	10	15	20
TA-PGMA	2	2.20 ×10^4	1.30					
TA-PGEA (TP)				0.32				
TA-PGEA-Gd(TP-Gd)				8.10				
TP/miRNA					0.18	0.17	0.14	0.15
TP-Gd/miRNA					0.14	0.17	0.15	0.15
PEI/miRNA					0.28	0.26	0.17	0.16
TP-Gd/miRNA-ColIV						0.15		

[a]Determined from GPC results. PDI = weight average molecular weight/number average molecular weight, or M_w/M_n.
[b]Determined from ICP-MS results.
[c]Determined from DLS results. PDI = Polydispersity Index.

Supplementary Table 2 | Primers used in this work.

Gene	Forward primer	Reverse primer
α-SMA	5'-GTCCCAGACATCAGGGAGTAA-3'	5'-TCGGATACTTCAGCGTCAGGA-3'
SM22α	5'-CAACAAGGGTCCATCCTACGG-3'	5'-ATCTGGGCGGCCTACATCA-3'
myh11	5'-AAGCTGCGGCTAGAGGTCA-3'	5'-CCCTCCCTTTGATGGCTGAG-3'
GADPH	5'-CATGGCCTTCCGTGTTCCTA-3'	5'-GCGGCACGTCAGATCCA-3'

Supplementary Table 3 | Different average Ct values for all the groups

Samples	Ct Values (miR-145)[a]	Ct Values (U6)[a]
TP-Gd/miR-neg	22.1972	22.7861
TP-Gd/miR-145	21.9111	23.0630
TP-Gd/miR-neg-ColIV	23.0042	23.6699
TP-Gd/miR-145-ColIV	21.5252	23.5769
PEI/miR-neg	21.3198	20.8528
PEI/miR-145	19.3692	19.3875

[a]Determined from PCR results.

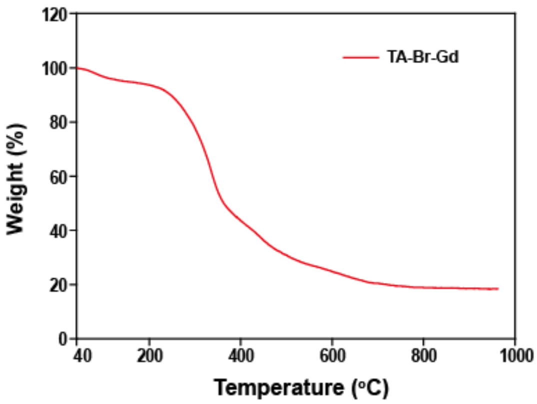

Supplementary Fig. 1 | Thermogravimetric data of TA-Br-Gd

Analysis: From the data, while the chelation was in a stable situation, the content of gadolinium accounted for nearly 20% of the total initiator. (Source data are provided as a Source Data file)

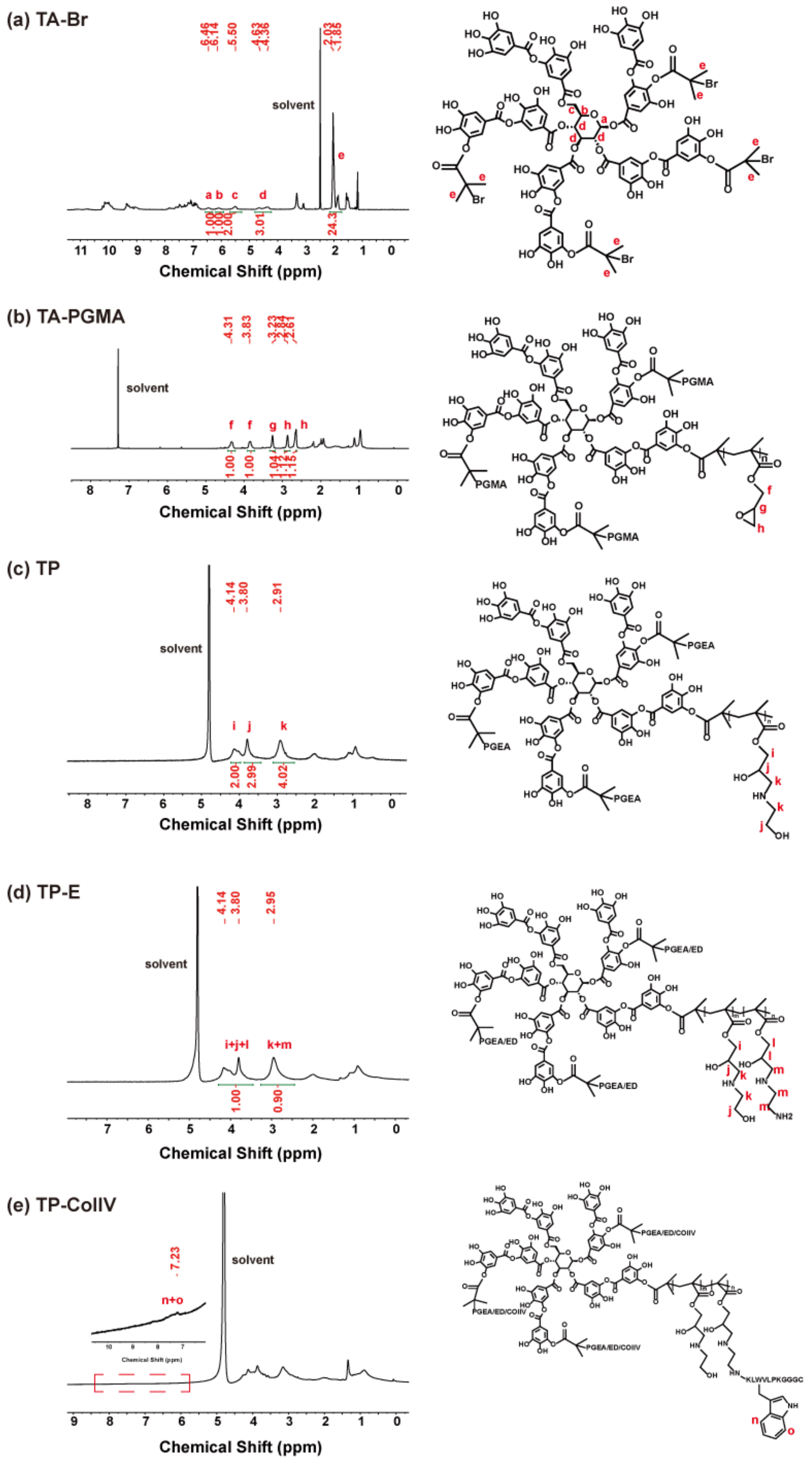

Supplementary Fig. 2 | ^{1}H NMR spectra of **a,** TA-Br, **b**, TA-PGMA, **c**, TP, **d**, TP-E and **e**, TP-ColIV.

Analysis: The chemical structures of TA-Br, TA-PGMA, TP, TP-E and TP-ColIV were characterized by ^{1}H NMR (Supplementary Fig. 2). For the NMR spectrum of TA-Br (Supplementary Fig. 2a), the peak at 6.46 ppm was associated with the methylidyne proton adjacent to the oxygen and oxygen moieties of the ester linkages (a, O=C-O-CH(O)-CH). The signal ζ = 6.14 ppm belonged to the methylidyne proton (b, CH_2-CH(O)-CH) and the peak at 5.50 ppm was related to the methylene protons (c, O-CH_2-CH). The CH-CH(O)-CH protons (d) related with the methylidyne protons of TA could be observed around 4.63 and 4.16 ppm. As expected, the area ratio of these peaks (a: b: c: d) was about 1:1:2:3, indicating the stable structure of tannic acid. The CH_3-C-Br protons (e) related with the bromoisobutyryl groups of synthetic TA-Br could be found around 2.03 and 1.85 ppm. The spectroscopic characterization data of TA-Br is consistent with the earlier report.[1] Based on the area ratio of peak a and peak e of TA-Br, about four phenolic hydroxyls of TA were reacted with BIBB.

TA-PGMA was prepared via ATRP based on TA-Br. As shown in Supplementary Fig. 2b, the peaks at δ = 3.23 (g), 2.84 and 2.61 (f) ppm were assigned to the protons of the epoxide ring in TA-PGMA. The area ratio of peaks f, g and h was approximately 2:1:2, manifesting that TA-PGMA maintains the integrity of the epoxy groups during ATRP.

The well-defined TA-PGMA was further modified by EA to produce the corresponding TA-PGEA (TP) for gene delivery. As shown in Supplementary Fig. 2c, some typical peaks of TA-PGMA disappeared after the ring-opening reaction. Three new peaks i (δ = 4.14 ppm), j (δ = 3.80 ppm) and k (δ = 2.91 ppm) appeared and the ratio area of peaks i and j to k was 5:4. The results indicated the successful and complete conversion from TA-PGMA to TP.

TP-E with primary amino groups was synthesized for coupling targeted peptide ColIV. TA-PGMA was modified with a certain proportion of EA and ED. As shown in Supplementary Fig. 2d, the ratio area of two new peaks for ED (l and m) was 3:6, which was different from the ratio in TP. Assuming that the amount of EA is x and the amount of ED is y, the computation formula was designed as follows and the ratio of EA and ED introduced in TP-E was finally calculated to be 7:1.

$$\begin{cases} 5x + 3y = 1 \\ 4x + 6y = 0.9 \end{cases} \tag{1}$$

TP-ColIV was synthesized via amidation reaction by the primary amino groups of TP-E and the carboxyl group of ColIV. As shown in Supplementary Fig. 2e, two new peaks n and o (δ = 7.23 ppm) appeared which represented the methylidyne protons of tryptophan in targeted peptide ColIV KLWVLPKGGGC, indicating the successful synthesis.

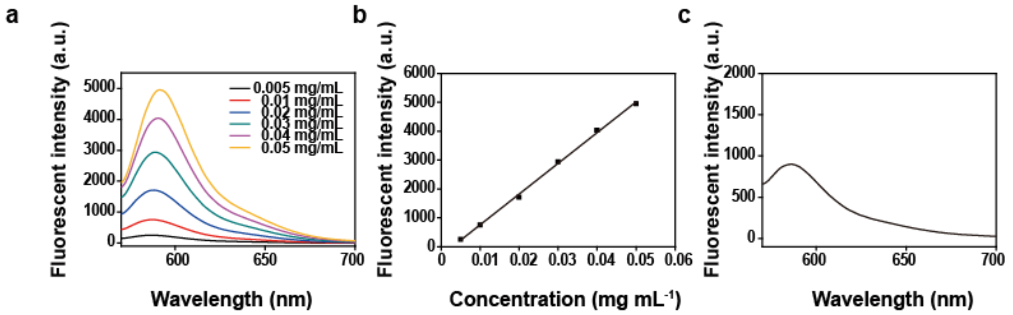

Supplementary Fig. 3 | The amount of grafted targeting peptide was calculated by fluorescent intensity. **a**, Fluorescent intensity of rhodamine B modified ColIV at different concentrations. **b**, A linear fitting of the fluorescent intensity and concentration of rhodamine B modified ColIV. **c**, Fluorescent intensity curve of TP-ColIV-RhB at the concentration of 0.04 mg mL^{-1}. (Source data are provided as a Source Data file)

Analysis: Rhodamine B modified targeted peptide ColIV was used to evaluate the reaction efficiency of targeting peptide. As shown in Supplementary Fig. 3a, the emission spectra of rhodamine B modified ColIV at different concentrations were detected by the excitation wavelength 550 nm. Thus, the maximum fluorescent intensity (y) at various concentrations (x) of rhodamine B modified ColIV by linear fitting was shown in Supplementary Fig. 3b and the relevant formula was obtained as follow:

$$y = 106458x - 309 \quad (2)$$

Also, the maximum fluorescent intensity of TP-ColIV-RhB at the concentration of 0.04 mg mL^{-1} from Supplementary Fig. S3c was 900.5. The concentration of rhodamine B modified targeted peptide ColIV could be calculated from the above formula to be 0.0114 mg mL^{-1}. Based on the molecular weights of TP-E (from TA-PGMA) and ColIV-RhB, approximately eight targeted peptides were introduced to one TP-E.

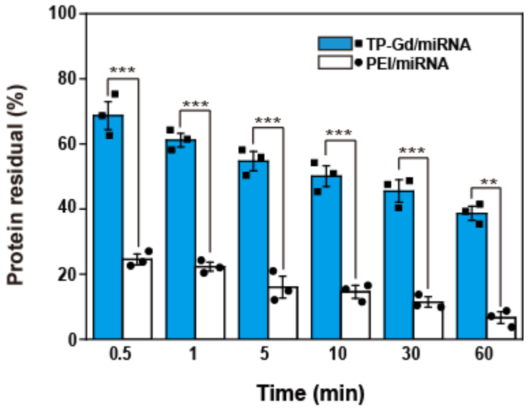

Supplementary Fig. 4 | Protein assay of TP-Gd/miRNA and PEI/miRNA complexes treated with excess bovine serum albumin (BSA). (**p < 0.01 and ***p < 0.001, see "Statistical analysis" in "Methods"; n = 3 independent experiments; error bars represent standard deviation; source data are provided as a Source Data file)

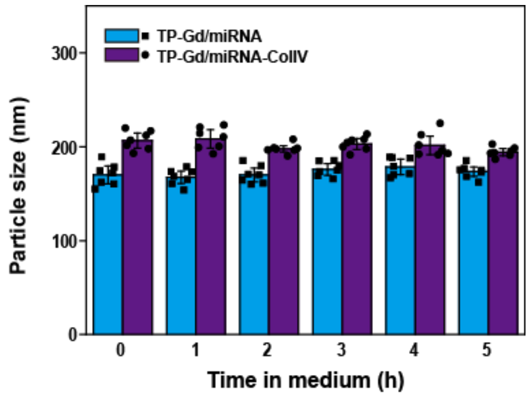

Supplementary Fig. 5 | Particle size stabilities of TP-Gd/miRNA and TP-Gd/miRNA-ColIV at the N/P ratio of 10 in medium with 10% FBS. (n = 7 independent experiments; error bars represent standard deviation; source data are provided as a Source Data file)

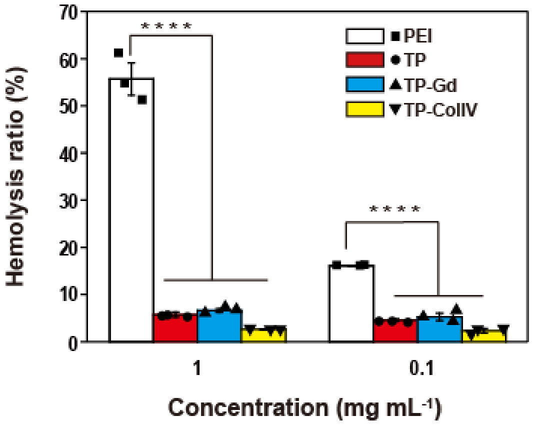

Supplementary Fig. 6 | Hemolysis ratio of RBCs treated with PEI, TP, TP-Gd and TP-ColIV at the concentrations of 1 mg mL^{-1} and 0.1 mg mL^{-1}. (****$p < 0.0001$, see "Statistical analysis" in "Methods"; n = 3 independent experiments; error bars represent standard deviation; source data are provided as a Source Data file)

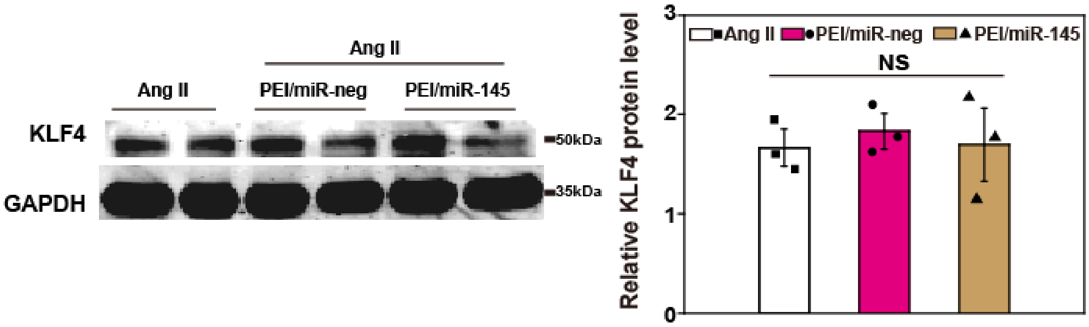

Supplementary Fig. 7 | Relative KLF4 protein expressions of PEI/miRNA complexes at the N/P ratio of 10 in SMCs. (n = 3 independent experiments; error bars represent standard deviation; source data are provided as a Source Data file)

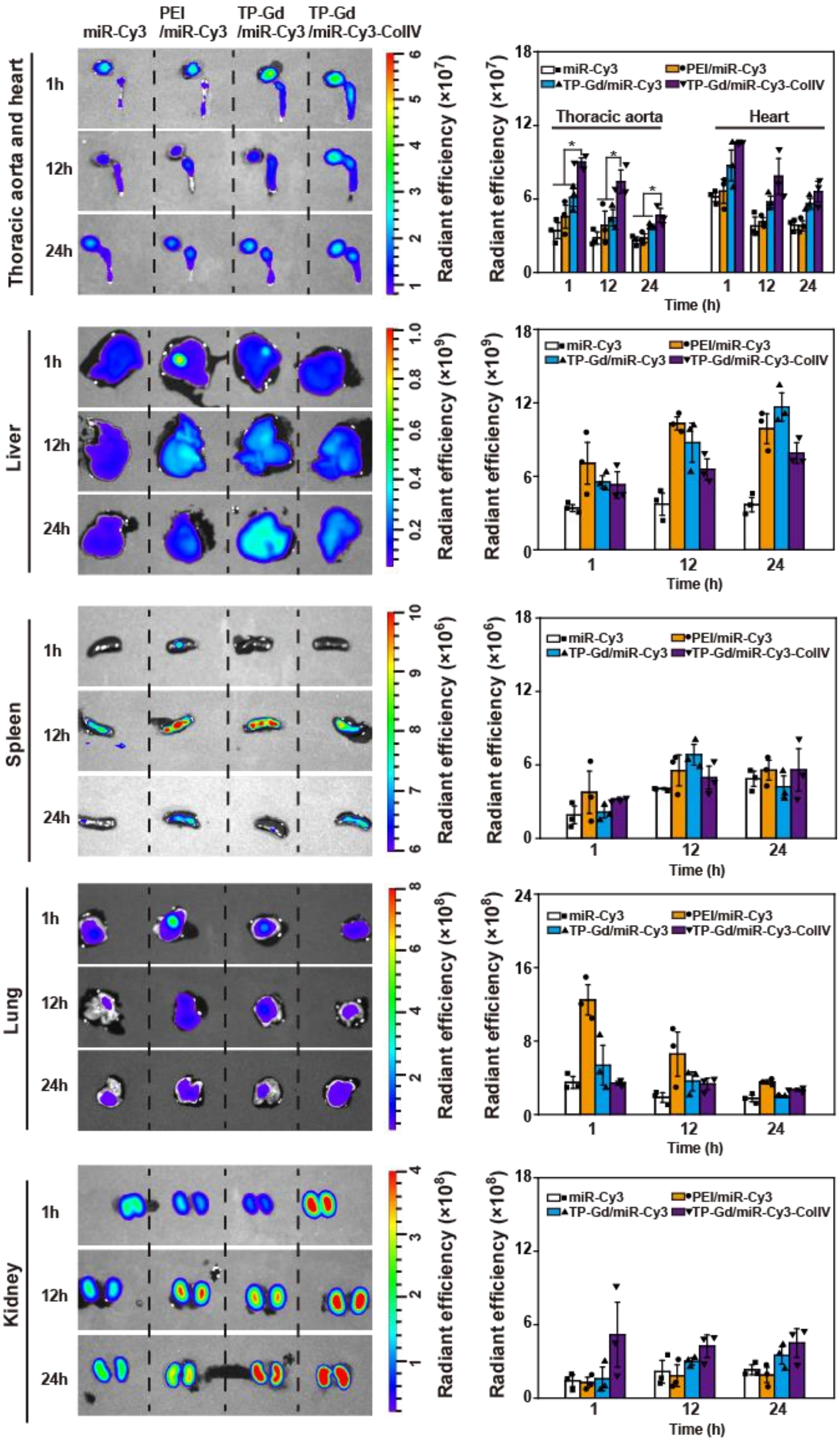

Supplementary Fig. 8 | Representative images and radiant efficiencies of all the organs after the accumulation of miR-Cy3 and polycation/miR-Cy3 complexes at different time points determined by Xenogen IVIS imaging system. (*$p < 0.05$, see "Statistical analysis" in "Methods"; n = 3 animals; error bars represent standard deviation; source data are provided as a Source Data file)

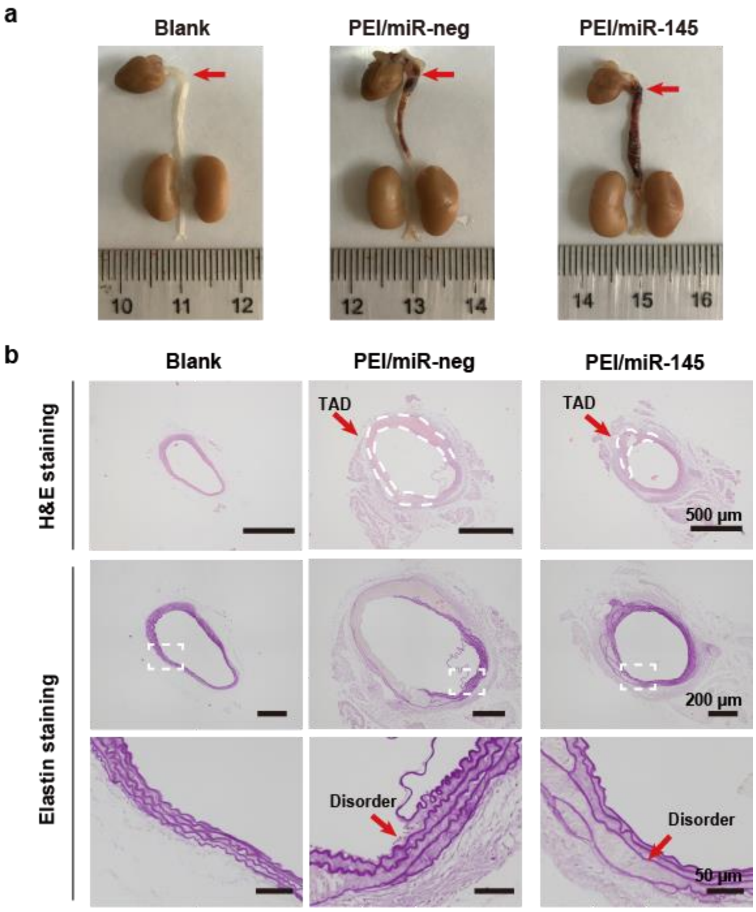

Supplementary Fig. 9 | *In vivo* effects of PEI/miR-145 complexes on BAPN-induced TAD model: **a**, general phenotypic pattern captured by digital camera and **b**, representative images of H&E- and elastin- thoracic aortic arches from the treated mice and healthy mice (blank).

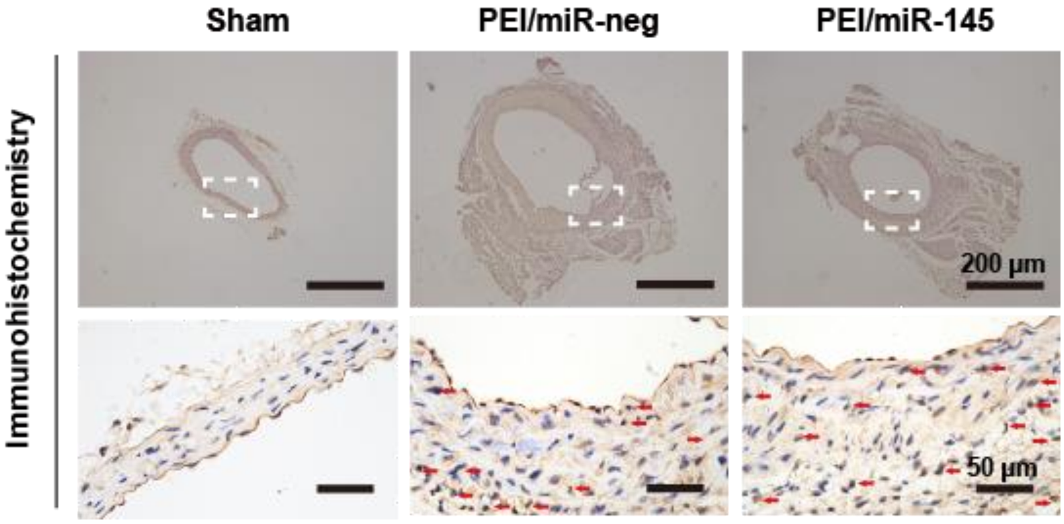

Supplementary Fig. 10 | Representative images of KLF4-positive immunohistochemical-stained thoracic aortic arches from the mice treated with PEI/miRNA complexes (healthy mice were set as control and red arrows highlighted positive areas).

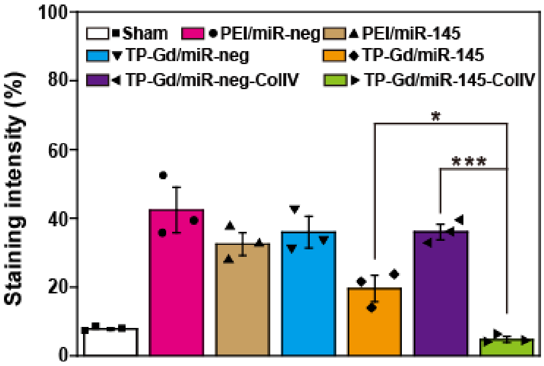

Supplementary Fig. 11 | Staining intensity of KLF4 for blank and polycation/miRNA complexes groups. (*p < 0.05 and ***p < 0.005, see "Statistical analysis" in "Methods"; n = 3 independent experiments; error bars represent standard deviation; source data are provided as a Source Data file)

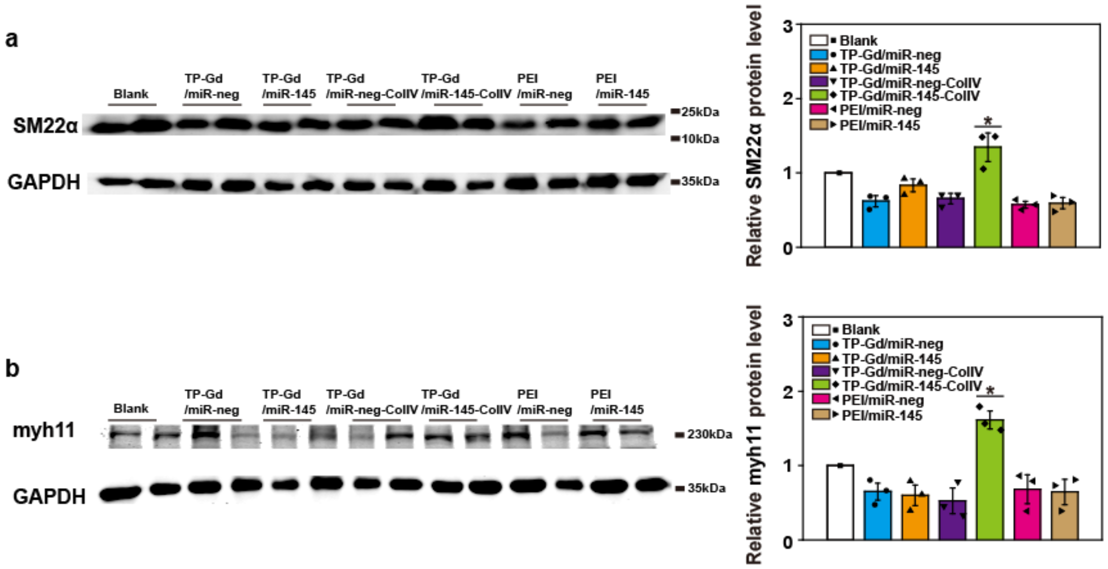

Supplementary Fig. 12 | *In vivo* relative SM22α (**a**) and myh11 (**b**) protein expressions determined by western blot. (**p* < 0.05, see "Statistical analysis" in "Methods"; n = 3 independent experiments; error bars represent standard deviation; source data are provided as a Source Data file)

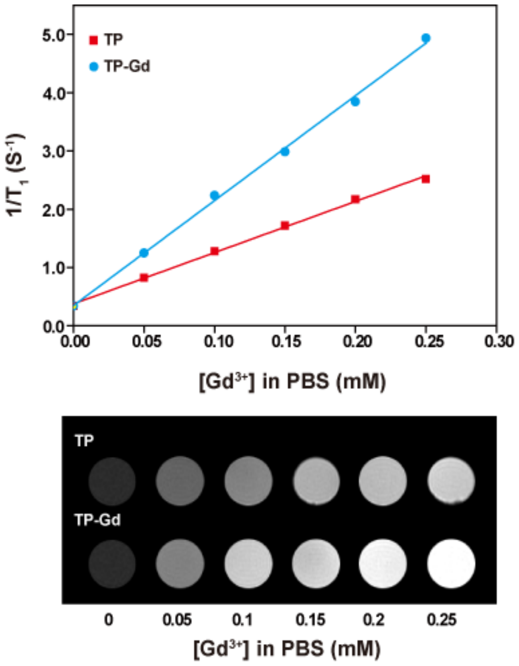

Supplementary Fig. 13 | Linear fitting of inverse T_1 (Source data are provided as a Source Data file) and T_1-weighted MR images of TP and TP-Gd in PBS under different Gd^{3+} concentrations.

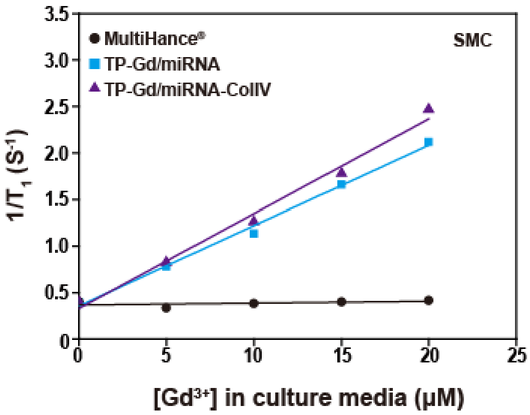

Supplementary Fig. 14 | Linear fitting of the inverse T_1 of MultiHance®, TP-Gd/miRNA and TP-Gd/miRNA-ColIV in SMCs under different Gd^{3+} concentrations. (Source data are provided as a Source Data file)

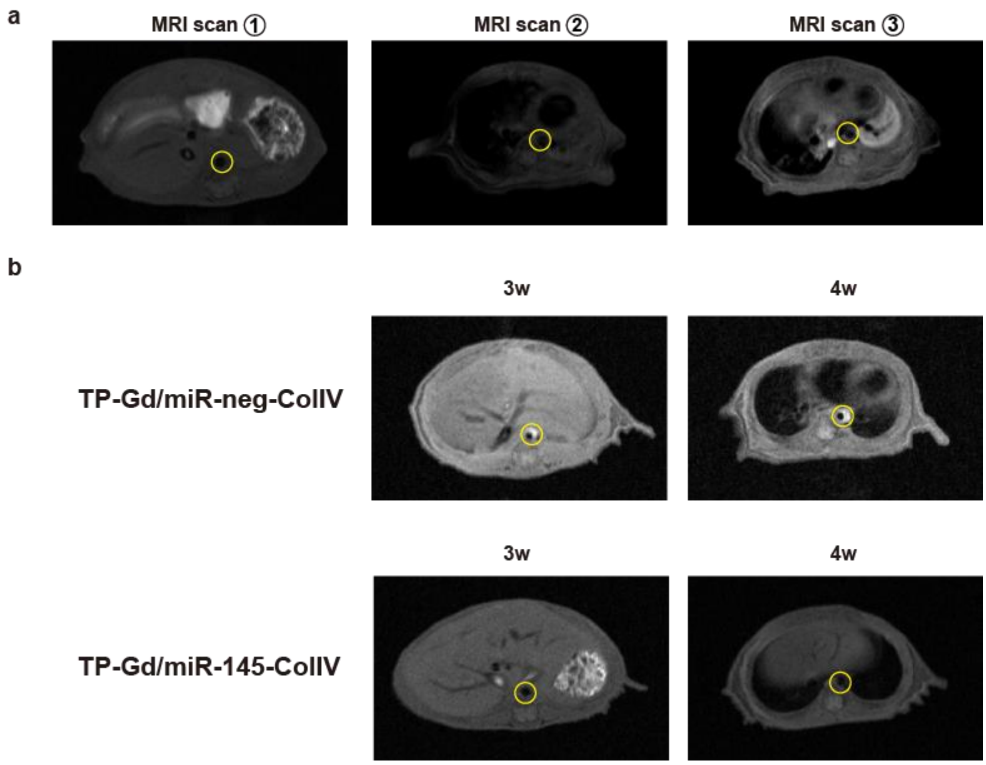

Supplementary Fig. 15 | Lower magnified MR images for **a**, diagnosis and **b**, noninvasive monitoring on BAPN-induced TAD model (yellow circles represent the detected area).

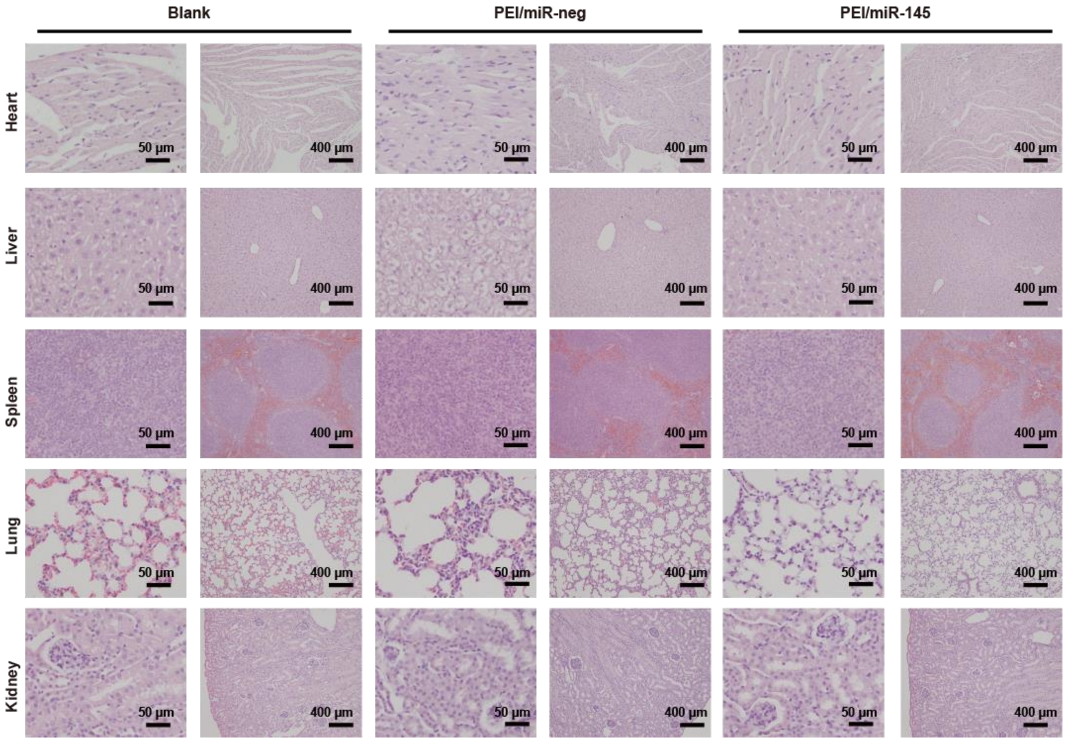

Supplementary Fig. 16 | Representative photographs of H&E staining of paraffin-embedded sections of different organs in the blank and PEI/miRNA groups.

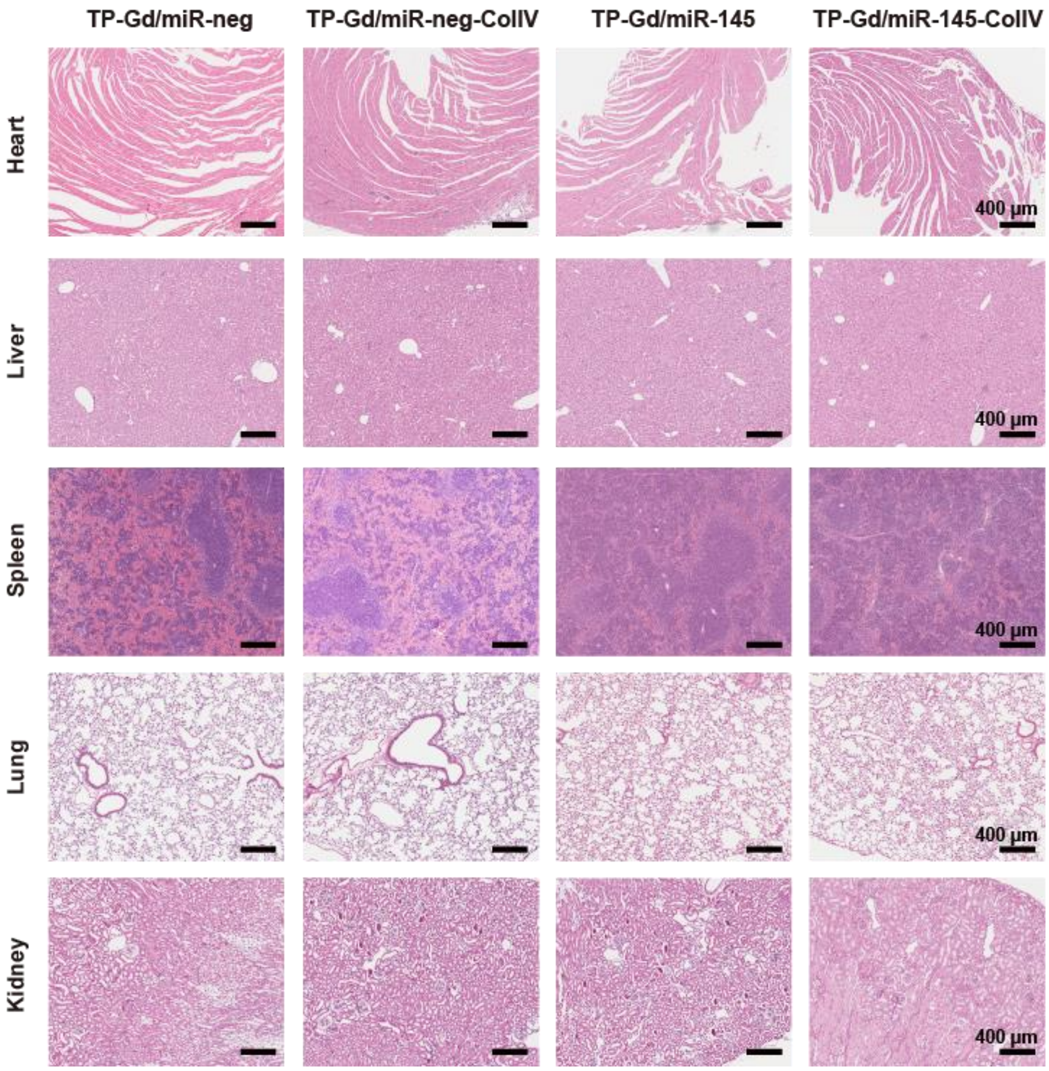

Supplementary Fig. 17 | Lower magnified photographs of H&E staining of paraffin-embedded sections of different organs in TP-containing polycation/miRNA groups.

References

1 Pranantyo, D. *et al.* Tea Stains-Inspired Initiator Primer for Surface Grafting of Antifouling and Antimicrobial Polymer Brush Coatings. Biomacromolecules **16**, 723-732 (2015).
